# Prediction of anastomotic leakage after esophagectomy for esophageal cancer: a nomogram study integrating systemic inflammation indices and clinical factors

**DOI:** 10.3389/fonc.2026.1821694

**Published:** 2026-07-14

**Authors:** Ruonan Tan, Lili Guo, Saitian Li, Weiran Huang, Hang Zhang, Tongtong Gu, Qian Ba

**Affiliations:** 1 Science and Technology Innovation Center, Shanghai Municipal Hospital of Traditional Chinese Medicine, Shanghai University of Traditional Chinese Medicine, Shanghai, China; 2 Department of Anesthesiology, Changzheng Hospital, Second Affiliated Hospital of Naval Medical University, Shanghai, China; 3 Department of Cardiothoracic Surgery, Huashan Hospital of Fudan University, Shanghai, China; 4 Qing Yuan Research Institute, School of Electronic Information and Electrical Engineering, Shanghai Jiao Tong University, Shanghai, China; 5 Department of Thoracic Surgery, Shanghai General Hospital, Shanghai Jiao Tong University School of Medicine, Shanghai, China; 6 Department of Pharmacy, Shanghai Sixth People’s Hospital Affiliated to Shanghai Jiao Tong University School of Medicine, Shanghai, China

**Keywords:** anastomotic leakage, esophageal cancer, inflammation indices, nomogram, predictive model

## Abstract

**Background:**

Esophageal cancer remains one of the leading causes of cancer-related mortality worldwide. Anastomotic leakage (AL) following esophagectomy is a major postoperative complication that significantly impacts patient outcomes, including mortality, morbidity, prolonged hospital stays, and increased healthcare costs. Despite advances in surgical techniques and adjuvant therapies, predicting the risk of AL remains a challenge.

**Objective:**

This study aims to develop and validate a predictive model for assessing the risk of AL in esophageal cancer patients undergoing esophagectomy, based on comprehensive clinical and laboratory variables.

**Methods:**

This retrospective cohort study included 650 esophageal cancer patients who underwent esophagectomy between January 2015 and May 2025, divided into a training set (n = 455) and a validation set (n = 195) at 7:3 ratio. Baseline demographic, clinicopathological, and laboratory data were collected, with AL as the primary outcome, defined according to the Esophagectomy Complications Consensus Group (ECCG). Univariable and multivariable logistic regression, restricted cubic splines (RCS), and nomogram development to identify predictors, with model performance assessed using receiver operating characteristic (ROC) curve, calibration plots, and decision curve analysis (DCA).

**Results:**

Seven significant predictors of AL were identified in the training set: age, neoadjuvant radiotherapy, C-reactive protein-albumin-lymphocyte (CALLY) index, hypertension, neutrophil-to-lymphocyte ratio (NLR), neutrophil-to-monocyte ratio (NMR), and platelet-to-lymphocyte ratio (PLR). A nomogram model was developed, showing good discrimination (AUC = 0.813) and calibration in the training set. The validation cohort demonstrated moderate predictive accuracy (AUC = 0.763), with consistent net benefits observed across different risk thresholds in DCA.

**Conclusions:**

In conclusion, this study established a potentially useful predictive model for AL risk, which may facilitate individualized risk stratification, guide perioperative decision-making, and ultimately contribute to reducing AL incidence and improving postoperative recovery.

## 1 Introduction

Esophageal carcinoma continues to rank among the principal contributors to global cancer-related deaths (1, 2). Although surgical methods and supportive treatments have progressed, anastomotic leakage (AL) remains a particularly serious complication after esophagectomy, substantially affecting patient morbidity, mortality rates, and medical expenditures (3, 4). AL develops when the surgically created junction between the residual esophagus and the stomach or colon becomes compromised, permitting gastrointestinal contents to escape into the mediastinal space or surrounding tissues. This can result in severe consequences, including localized or systemic infection, sepsis, and extended hospitalization (5–7). AL is associated with increased mortality, additional surgical interventions, impaired quality of life, and substantially higher healthcare costs (8, 9). These consequences underscore the urgent need for reliable predictive tools and effective preventive strategies in esophageal cancer management.

The incidence of AL after esophagectomy varies considerably, ranging from 5% to 30% depending on the definition criteria, surgical technique, and patient population (10). This wide variation reflects differences in AL grading systems, with the Esophagectomy Complications Consensus Group (ECCG) defining AL as a full-thickness gastrointestinal defect involving the esophagus, anastomosis, staple line, or conduit, irrespective of presentation or method of identification (11). Several factors have been identified as potential risk factors for AL, including patient demographics, tumor characteristics, surgical variables, and postoperative inflammatory markers (12). Specifically, advanced age, male sex, malnutrition, diabetes, and prolonged operative time have been consistently associated with increased AL risk (13, 14). Additionally, emerging evidence suggests that systemic inflammatory markers—such as C-reactive protein (CRP), neutrophil-to-lymphocyte ratio (NLR), and platelet-to-lymphocyte ratio (PLR)—may serve as valuable predictors of postoperative complications (15, 16).

Recently, composite inflammatory-nutritional indices have gained attention for their ability to integrate multiple pathophysiological processes. The C-reactive protein-albumin-lymphocyte (CALLY) index, combining CRP, albumin, and lymphocyte count, has demonstrated prognostic value in various malignancies (17). Similarly, the CRP-to-albumin ratio (CAR) and systemic immune-inflammation index (SII) have shown promise in predicting surgical outcomes (18). However, the utility of these composite indices in predicting AL after esophagectomy remains underexplored.

Minimally invasive surgical techniques, including laparoscopic and thoracoscopic approaches, have been increasingly adopted in esophageal cancer surgery. While some studies suggest that minimally invasive esophagectomy (MIE) may reduce surgical trauma and improve outcomes, its impact on AL risk remains controversial (19). The challenge lies in effectively predicting which patients are at a higher risk for AL, as this would allow for better preoperative optimization, personalized treatment strategies, and targeted interventions.

Nomograms have emerged as valuable tools for individualized risk prediction in oncologic surgery. By integrating multiple risk factors, nomograms provide visual, user-friendly interfaces for estimating patient-specific probabilities of adverse outcomes (20). Several nomograms have been developed to predict complications after colorectal and gastric cancer surgery (21); however, few studies have focused specifically on AL prediction after esophagectomy, and none have incorporated the comprehensive panel of inflammatory-nutritional indices evaluated in the present study.In this study, we aim to develop and validate a predictive model for AL risk in esophageal cancer patients undergoing esophagectomy. By utilizing clinical and laboratory data from a large cohort of patients, we applied advanced statistical techniques, including logistic regression, restricted cubic splines (RCS), and nomogram development, to identify independent predictors of AL. We also assessed the performance and clinical utility of the predictive model using both training and validation cohorts. The findings from this study may offer valuable insights into improving risk stratification for esophageal cancer patients and guide clinical decision-making regarding perioperative management.

## 2 Materials and methods

### 2.1 Study design and participants

This retrospective cohort study involved esophageal cancer patients who underwent esophagectomy at Shanghai General Hospital and Huashan Hospital of Fudan University from January 2015 to May 2025. The inclusion criteria were (1): Histologically confirmed esophageal cancer diagnosed by preoperative gastroscopic biopsy (2); patients deemed eligible for curative esophagectomy. Exclusion criteria were (1): history of prior cancer surgery or other malignancies (2); incomplete clinical data (3); emergency surgery for intestinal obstruction or hemorrhage. The study adhered to the Declaration of Helsinki and received approval from the institutional ethics committees. All patients provided written informed consent.

### 2.2 Definition of AL

Clinical AL was defined as the leakage of gastrointestinal digestive juice resulting from the loss of integrity of the surgical anastomosis based on the criteria proposed by the ECCG (3). The diagnosis was confirmed by clinical symptoms (fever, tachycardia, chest pain, or sepsis) combined with imaging (contrast swallow or computed tomography) or endoscopic findings demonstrating extravasation of contrast or visible defect at the Anastomotic site.

### 2.3 Data collection

Categorical variables were presented as frequencies and percentages and compared using the chi-square test or Fisher’s exact test, as appropriate. Continuous variables were expressed as mean ± standard deviation (SD) for normally distributed data and median (interquartile range, IQR) for non-normally distributed data. Normality was assessed using the Shapiro–Wilk test. Comparisons between groups were performed using the Student’s t-test or Mann–Whitney U test, as appropriate. After exclusion of patients with incomplete data, no missing values remained in the final analytical dataset. Patient-related (age, sex, BMI, hypertension, cardiac function), tumor-related (TNM stage, tumor location, histological type, differentiation, neoadjuvant therapy, nerve invasion, vascular tumor thrombus), surgery-related (operation time, laparoscopic surgery, surgical approach), laboratory variables (albumin, leukocyte, monocyte, platelet, urea, C-reactive, Ki67, and inflammatory-nutritional composite indices [NLR, PLR, NMR, CAR, SII, and CALLY index]) were retrospectively collected from medical records and considered as potential risk factors for AL. AL is a multifactorial complication. Six inflammatory and inflammation-related indices—NLR, PLR, NMR, SII, CAR, and CALLY were selected *a priori* based on their established clinical relevance and biological plausibility in perioperative risk assessment. These indices reflect complementary aspects of tumor-related inflammation, including immune balance, platelet-mediated inflammation, systemic inflammatory burden, acute-phase response, and nutrition–inflammation interaction. Laboratory variables and inflammatory-nutritional composite indices were obtained on the morning of the second day after hospital admission, prior to surgery.

### 2.4 Analysis using logistic regression

To assess the associations between various predictor variables and AL, both univariable and multivariable logistic regression models were employed. Initially, univariable analysis was conducted to evaluate the individual relationships between each predictor variable and the outcome. Variables that demonstrated statistical significance at the p < 0.1 level in the univariable analysis were then included in the multivariable logistic regression model to control for potential confounders. Multicollinearity among the predictor variables was assessed using the variance inflation factors (VIFs), predictors with VIF > 10 were removed from the final model. Sensitivity analyses were performed using Firth penalized logistic regression to reduce potential small-sample bias and separation. Calendar year was additionally included as a covariate to account for potential temporal effects. Penalized odds ratios (ORs) and 95% confidence intervals (CIs) were calculated.

### 2.5 Restricted cubic splines

To examine the nonlinear relationship between continuous predictor variables and the outcome (AL), we performed RCS analysis using the rms package in R. Continuous predictors were modeled with four knots placed at 5th, 35th, 65th, and 95th percentiles to capture nonlinear effects. Binary variables (neoadjuvant radiotherapy and hypertension) were included as covariates but not transformed. The model was adjusted for potential confounders, including demographic and clinical variables. Nonlinearity was assessed by the likelihood ratio test comparing the RCS model with a linear model; a P value < 0.05 indicated significant nonlinearity.

### 2.6 Development of the nomogram

A nomogram model was developed using independent predictors from multivariable logistic regression to estimate the likelihood of postoperative AL. Each variable received a weighted score based on its regression coefficient, with the total score representing the predicted probability ranging from 0 to 1.

### 2.7 Model validation and performance assessment

The predictive performance of nomogram was assessed in both training and validation cohorts. The model’s discriminative ability was quantified using the concordance index (C-index), which is equivalent to the area under the ROC curve. Moreover, ROC-derived cutoff values were calculated for continuous predictors included in the nomogram using the maximum Youden index. For additional risk stratification, the total nomogram score was calculated for each patient and categorized into tertiles to define low-, intermediate-, and high-risk groups. Calibration was assessed using calibration plots, the Hosmer–Lemeshow goodness-of-fit test, and the Brier score. Internal validation was performed using bootstrap resampling with 1,000 iterations to correct for overfitting bias. Clinical utility was evaluated by decision curve analysis (DCA), which quantifies the net benefit across a range of threshold probabilities by comparing the nomogram-guided strategy with “treat-all” and “treat-none” strategies.

All statistical analyses were conducted in R software (version 4.4.1). Logistic regression analyses were performed using the “stats” package, and Firth penalized logistic regression was fitted with “logistf”. The “rms” package was used for restricted cubic spline analysis, nomogram construction, and calibration assessment. ROC analysis and cutoff value determination were conducted using “pROC”, whereas decision curve analysis was performed with “rmda”. Figures were generated using “ggplot2”. All statistical tests were two-sided, with P < 0.05 considered statistically significant.

## 3 Results

### 3.1 Selection of cases and initial clinical characteristics

**Figure 1:**
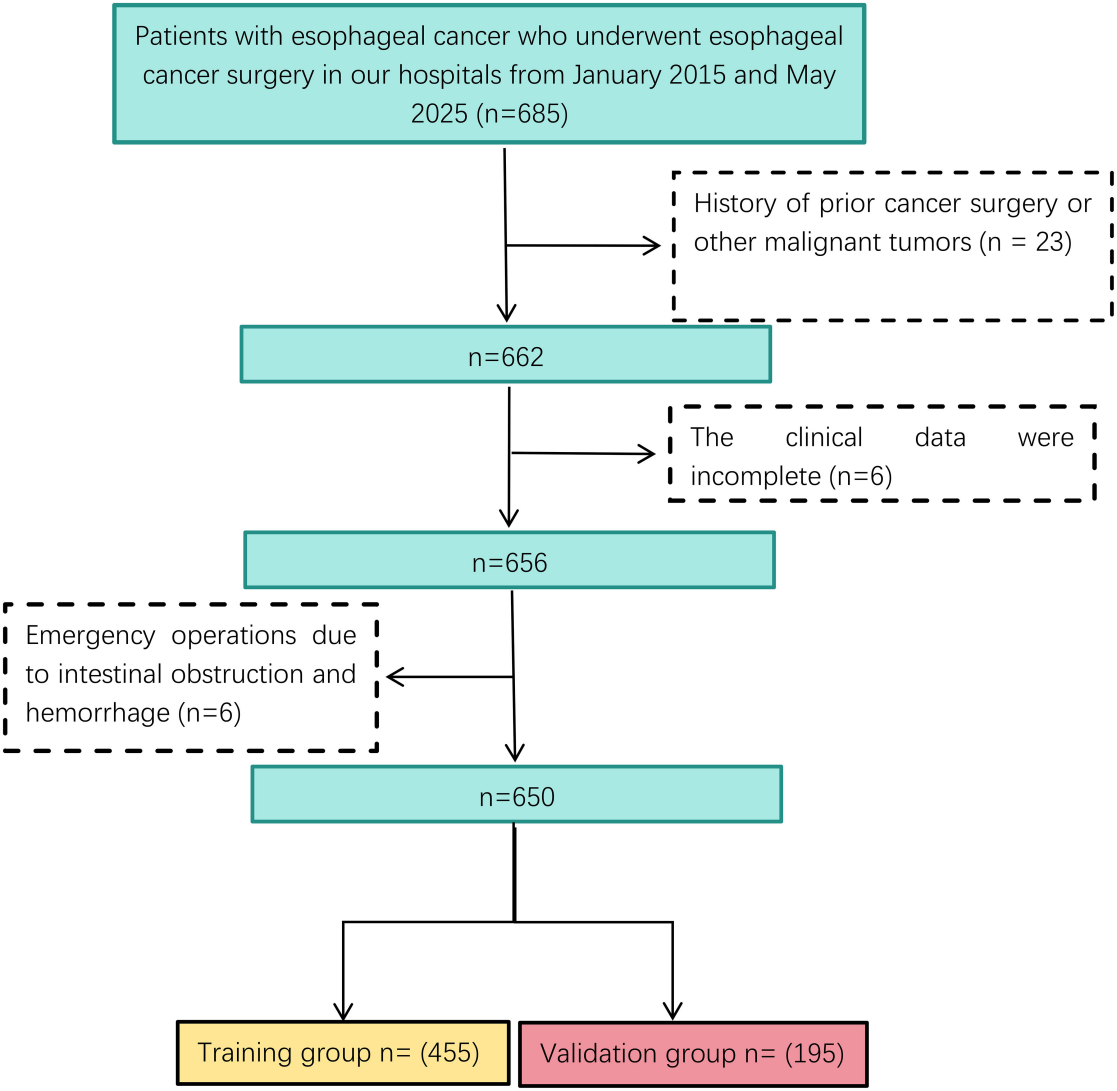
Flowchart of patient selection. A total of 685 patients undergoing esophagectomy were screened. After exclusions for prior malignancy, incomplete data, and emergency surgery, 650 eligible patients were included and randomly allocated to the training (n=455) and validation (n=195) cohorts.

A total of 685 patients with esophageal cancer who underwent esophagectomy at our hospitals between January 2015 and May 2025 were initially screened. After excluding individuals with a history of malignancy or previous cancer surgeries (n=23), with incomplete data (n=6), and who required emergency surgery (n=6), 650 patients remained in the study. Among these, 455 patients were included in the training group, while 195 were included in the validation group at a 7:3 ratio (**Figure 1**). A correlation heatmap of clinicopathological and laboratory variables is shown in (**Supplementary Figure 1**) to evaluate potential multicollinearity among predictors and to explore preliminary associations with AL. Clustering of inflammatory indices, hematologic parameters, and tumor staging variables was observed. **Table 1** provides a summary of the baseline clinical characteristics. In the training cohort, significant differences were observed between patients with AL and non-AL in sex, age, BMI, hypertension, neoadjuvant radiotherapy and laparoscopic surgery (all P < 0.05). AL patients exhibited significantly higher NLR, PLR, CRP and CAR, but lower albumin, CALLY index, and NMR values (all P < 0.01). In contrast, most tumor-related and pathological variables—such as tumor location, TNM stage, degree of differentiation, nerve invasion, vascular tumor thrombus, and Ki-67 index—did not differ significantly between groups. Similar patterns were observed in the validation cohort, where inflammatory and nutritional indicators, particularly CALLY, NLR, SII, NMR, CAR and CRP, remained significantly associated with AL, whereas tumor characteristics showed no significant differences.

**Table 1:** Baseline clinical characteristics of the patients in the training and validation cohort.

Characteristics	Training group (N = 455)		P value	Validation group (N = 195)		P value
	N-AL (n=350)	AL (n=105)		N-AL (n=147)	AL (n=48)	
Sex, n (%)			< 0.001			0.859
Female	60 (17.14%)	41 (39.05%)		23 (15.65%)	7 (14.58%)	
Male	290 (82.86%)	64 (60.95%)		124 (84.35%)	41 (85.42%)	
Age, median (IQR)	65.00 (59.25 – 70.00)	66.00 (61.00 – 71.00)	**< 0.001**	66.00 (60.00 – 71.00)	66.00 (64.00 – 73.00)	0.211
Year, n (%)			**0.015**			**< 0.001**
2015-2020	94 (26.86%)	42 (52.38%)		54 (36.73%)	5 (10.42%)	
2020-2025	256 (73.14%)	63 (47.62%)		93 (63.27%)	43 (89.58%)	
BMI, n (%)			< 0.001			0.165
18.5-24.5	266 (76.00%)	53 (50.48%)		87 (59.18%)	35 (72.92%)	
<18.5	32 (9.14%)	9 (8.57%)		11 (7.48%)	1 (2.08%)	
≧24.5	52 (14.86%)	43 (40.95%)		49 (33.34%)	12 (25.00%)	
Hypertension, n (%)			< 0.001			0.147
No	289 (82.57%)	30 (28.57%)		108 (73.47%)	30 (62.50%)	
Yes	61 (17.43%)	75 (71.43%)		39 (26.53%)	18 (37.50%)	
Cardiac function, median (IQR)	63 (62.00 – 66.00)	64 (62.00 – 66.00)	0.813	64 (62.00 – 66.00)	63 (62.00 – 66.00)	0.357
Neoadjuvant radiotherapy, n (%)			< 0.001			0.159
No	315 (90.00%)	53 (50.48%)		115 (78.23%)	42 (87.50%)	
yes	35 (10.00%)	52 (49.52%)		32 (21.77%)	6 (12.50%)	
Infarction, n (%)			< 0.001			0.859
NO	309 (88.29%)	92 (87.62%)		124 (84.35%)	41 (85.42%)	
Yes	41 (11.71%)	13 (12.38%)		23 (15.65%)	7 (14.58%)	
CALLY, median (IQR)	4.10 (2.06 – 9.03)	2.71 (2.70 – 6.57)	**< 0.001**	3.85 (0.98 – 10.12)	1.18 (0.41 – 3.89)	**< 0.001**
NLR, median (IQR)	1.95 (1.44 – 2.71)	4.18 (3.64 – 5.08)	**< 0.001**	2.15 (1.57 – 2.87)	2.68 (1.99 – 3.69)	**0.002**
PLR, median (IQR)	109.00 (80.35 – 151.18)	258.70 (233.00 – 312.30)	**< 0.001**	127.73 (97.34 – 168.21)	150.55 (101.56 – 203.84)	0.129
SII, median (IQR)	445.00 (315.25 – 653.00)	447.00 (274.00 – 641.00)	0.704	438.17 (301.72 – 632.59)	554.59 (376.95 – 799.01)	**0.019**
NMR, median (IQR)	10.01 (8.38 – 12.40)	5.84 (3.88 – 8.31)	**< 0.001**	8.61 (7.04 – 10.58)	9.32 (7.93 – 12.70)	**0.047**
CAR, median (IQR)	0.02 (0.00 – 0.13)	0.24 (0.20 – 0.37)	**< 0.001**	0.04 (0.02 – 0.15)	0.11 (0.03 – 0.35)	**< 0.001**
Lymphocyte, median (IQR)	1.55 (1.26 – 1.82)	1.43 (1.18 – 1.83)	0.209	1.62 (1.24 – 2.03)	1.42 (1.13 – 1.75)	**0.015**
Monocyte, median (IQR)	0.36 (0.29 – 0.47)	0.38 (0.30 – 0.49)	0.406	0.40 (0.31 – 0.49)	0.39 (0.33 – 0.57)	0.421
Platelet, median (IQR)	203.50 (158.25 – 242.50)	189.00 (154.00 – 240.00)	0.365	199.00 (161.00 – 257.50)	198.50 (155.75 – 263.00)	0.859
CRP, median (IQR)	0.50 (0.10 – 4.70)	13.00 (11.40 – 18.90)	**< 0.001**	1.50 (0.80 – 6.15)	4.60 (1.48 – 13.28)	**< 0.001**
Albumin, mean ± sd	41.28 ± 4.70	37.27 ± 4.34	**< 0.001**	40.88 ± 4.29	39.88 ± 3.80	0.149
Creatinine, median (IQR)	65.85 (58.83 – 78.00)	67.00 (59.00 – 71.60)	0.560	66.00 (57.15 – 74.65)	65.95 (59.60 – 75.20)	0.992
Urea, median (IQR)	5.42 (4.50 – 6.44)	4.90 (4.12 – 6.08)	**0.009**	5.78 (4.60 – 6.70)	5.15 (4.53 – 6.26)	0.280
Tumor location, n (%)			0.551			0.769
Thoracic	295 (84.26%)	91 (86.67%)		128 (87.07%)	41 (85.42%)	
Abdomen	55 (15.74%)	14 (13.33%)		19 (12.93%)	7 (14.58%)	
Tumor classification, n (%)			0.987			0.917
Squamous cell carcinoma	294 (84.00%)	88 (83.81%)		119 (80.95%)	40 (83.33%)	
Adenocarcinoma	47 (13.43%)	14 (13.33%)		20 (13.61%)	6 (12.50%)	
Other types	9 (2.57%)	3 (2.86%)		8 (5.44%)	2 (4.17%)	
Degree of differentiation, n (%)			0.632			0.147
0*	6 (1.71%)	1 (0.95%)		10 (6.81%)	0	
Poor	90 (25.71%)	32 (30.48%)		53 (36.05%)	16 (33.33%)	
Moderate	213 (60.86%)	63 (60.00%)		73 (49.66%)	25 (52.08%)	
Well	41 (11.72%)	9 (8.57%)		11 (7.48%)	7 (14.59%)	
T, n (%)			0.975			0.468
1	64 (18.29%)	19 (18.10%)		30 (15.4%)	7 (3.6%)	
2	82 (23.43%)	23 (21.90%)		45 (23.1%)	13 (6.7%)	
3	184 (52.57%)	56 (53.55%)		65 (33.3%)	27 (13.8%)	
4	20 (5.71%)	7 (6.67%)		7 (3.6%)	1 (0.5%)	
N, n (%)			0.872			0.534
0	205 (58.57%)	63 (60.00%)		90 (61.22%)	28 (58.33%)	
1	91 (26.00%)	24 (22.86%)		36 (24.49%)	16 (33.33%)	
2	39 (11.14%)	12 (11.43%)		14 (9.52%)	3 (6.25%)	
3	15 (4.29%)	6 (5.71%)		7 (4.77%)	1 (2.09%)	
M, n (%)			0.187			0.990
0	347 (99.14%)	103 (98.10%)		146 (99.32%)	47 (97.92%)	
1	3 (0.86%)	2 (1.90%)		1 (0.68%)	1 (2.08%)	
Stage, n (%)			0.986			0.568
I	61 (17.43%)	19 (18.10%)		29 (19.73%)	7 (14.58%)	
II	169 (48.29%)	51 (48.57%)		75 (51.02%)	28 (58.33%)	
III	89 (25.43%)	25 (23.81%)		30 (20.41%)	11 (22.92%)	
IV	31 (8.85%)	10 (9.52%)		13 (8.84%)	2 (4.17%)	
Nerve invasion, n (%)			0.748			0.254
No	226 (64.57%)	66 (62.86%)		94 (48.2%)	35 (72.92%)	
Yes	124 (35.43%)	39 (37.14%)		53 (27.2%)	13 (27.08%)	
Vascular tumor thrombus, n (%)			0.342			0.652
No	231 (66.00%)	64 (60.95%)		99 (67.35%)	34 (70.83%)	
Yes	119 (34.00%)	41 (39.05%)		48 (32.65%)	14 (29.17%)	
Ki67, median (IQR)	50.00 (30.00 – 70.00)	45.00 (30.00 – 70.00)	0.811	50.00 (30.00 – 60.00)	42.50 (30.00 – 70.00)	0.991
Operation time, median (IQR)	300.00 (240.00 – 370.00)	270.00 (230.00 – 340.00)	0.169	340.00 (260.00 – 410.00)	335.00 (257.50 – 421.25)	0.782
Surgical approach, n (%)			0.059			0.970
Lvor-Lewis	107 (30.57%)	20 (19.05%)		68 (46.26%)	22 (45.83%)	
McKeown	218 (62.29%)	78 (74.29%)		74 (50.34%)	24 (50.00%)	
Transhiatal esophagectomy	25 (7.14%)	7 (6.66%)		5 (3.40%)	2 (4.17%)	
Laparoscopic surgery, n (%)			0.014			0.040
No	303 (86.57%)	100 (95.24%)		135 (91.84%)	39 (81.25%)	
Yes	47 (13.43%)	5 (4.76%)		12 (8.16%)	9 (18.75%)	

*: 1) Patients with no residual tumor after neoadjuvant therapy; 2) Patients who underwent endoscopic submucosal dissection (ESD) for esophageal lesions (pathologically confirmed as malignant), followed by radical esophagectomy, with no residual tumor found in postoperative pathology. Bold values indicate statistical significance (P value < 0.05).

### 3.2 Identification of predictors for AL

The univariable and multivariable logistic regression results for AL were presented in **Table 2**. In the univariable analysis, significant predictors of AL included male (R = 0.323, 95%CI 0.200 – 0.523, P<0.001), older age (OR = 1.251, 95% CI 1.202–1.309), BMI ≥24.5 (OR = 4.150, 95% CI 2.519–6.862), hypertension (OR = 11.844, 95% CI 7.219–19.885), neoadjuvant radiotherapy (OR = 8.830, 95% CI 5.293–14.947), elevated NLR, PLR, CAR and CRP, as well as lower CALLY, NMR, and albumin levels. These variables were included in the multivariable model. In the multivariable analysis, after adjusting for confounders, age (OR = 1.319, 95% CI 1.161–1.585, P<0.001), hypertension (OR = 21.607, 95% CI 3.192–284.724, P = 0.005), and neoadjuvant radiotherapy (OR = 16.759, 95% CI 1.719–297.207, P = 0.026) remained statistically significant, though the latter two exhibited wide confidence intervals reflecting limited precision in effect estimation. Higher NLR (OR = 2.596, 95% CI 1.512–5.183, P = 0.002) and PLR (OR = 1.020, 95% CI 1.007–1.037, P = 0.007) were also significant, while NMR was protective (OR = 0.369, 95% CI 0.207–0.534, P<0.001). Other variables lost significance in the adjusted model. The VIFs for all predictor variables included in the model were below 10, indicating no significant multicollinearity in the multivariable logistic regression model (**Supplementary Table 1**).

**Table 2:** Univariable and multivariable logistic regression analyses of risk factors for AL.

Characteristics	Total(N)	Univariable analysis		Multivariable analysis	
		Odds ratio (95% CI)	P value	Odds ratio (95% CI)	P value
Sex	455				
Female	101	Reference			
Male	354	0.323 (0.200 – 0.523)	<0.001	0.426 (0.048 – 3.463)	0.424
Age	455	1.251 (1.202 – 1.309)	<0.001	1.319 (1.161 – 1.585)	<0.001
BMI	455				
18.5-24.5	266	Reference			
<18.5	32	1.412 (0.604 – 3.022)	0.396	1.990 (0.001 – 695.928)	0.849
≧24.5	52	4.150 (2.519 – 6.862)	<0.001	1.382 (0.186 – 10.083)	0.745
Hypertension	455				
No	319	Reference		Reference	
Yes	136	11.844 (7.219 – 19.885)	< 0.001	21.607 (3.192 – 284.724)	0.005
Cardiac function	455	1.010 (0.945 – 1.080)	0.767		
Infarction	455				
No	401	Reference			
Yes	54	1.065 (0.529 – 2.023)	0.853		
Neoadjuvant radiotherapy	455				
No	368	Reference		Reference	
Yes	87	8.830 (5.293 – 14.947)	< 0.001	16.759 (1.719 – 297.207)	0.026
CALLY	455	0.713 (0.634 – 0.790)	< 0.001	0.906 (0.814 – 0.999)	0.044
NLR	455	1.677 (1.466 – 1.938)	< 0.001	2.596 (1.512 – 5.183)	0.002
PLR	455	1.023 (1.018 – 1.027)	< 0.001	1.020 (1.007 – 1.037)	0.007
SII	455	1.000 (0.999 – 1.000)	0.732		
NMR	455	0.634 (0.571 – 0.698)	< 0.001	0.369 (0.207 – 0.534)	< 0.001
CAR	455	1.735 (1.219 – 2.570)	0.003	0.249 (0.018 – 2.031)	0.271
Lymphocyte	455	0.710 (0.453 – 1.093)	0.127		
Monocyte	455	1.678 (0.760 – 3.739)	0.185		
Platelet	455	0.999 (0.996 – 1.002)	0.581		
CRP	455	1.049 (1.033 – 1.067)	<0.001	1.006 (0.959 – 1.061)	0.802
Creatinine	455	0.994 (0.978 – 1.009)	0.449		
Albumin	455	0.825 (0.779 – 0.870)	<0.001	0.897 (0.718 – 1.095)	0.300
Urea	455	0.856 (0.741 – 0.981)	0.030	1.007 (0.572 – 1.697)	0.981
Tumor location	455				
Thoracic	386	Reference			
Abdomen	69	0.825 (0.424 – 1.515)	0.551		
Tumor classification	455				
Squamous cell carcinoma	382	Reference			
Adenocarcinoma	61	0.995 (0.507 – 1.849)	0.988		
Other types	12	1.114 (0.243 – 3.825)	0.874		
Degree of differentiation	455				
0*	7	Reference			
Poor	122	2.133 (0.346 – 41.108)	0.491		
Moderate	276	1.775 (0.296 – 33.846)	0.599		
Well	50	1.317 (0.190 – 26.475)	0.809		
T	455				
1	83	Reference			
2	105	0.945 (0.474 – 1.898)	0.872		
3	240	1.025 (0.574 – 1.890)	0.935		
4	27	1.179 (0.411 – 3.125)	0.747		
N	455				
0	268	Reference			
1	115	0.858 (0.498 – 1.445)	0.572		
2	51	1.001 (0.477 – 1.978)	0.997		
3	21	1.302 (0.449 – 3.351)	0.601		
M	455				
0	450	Reference			
1	5	0.701 (0.411 – 1.196)	0.193		
Stage	455				
I	80	Reference			
II	220	0.969 (0.537 – 1.801)	0.918		
III	114	0.902 (0.458 – 1.795)	0.766		
IV	41	1.036 (0.418 – 2.461)	0.938		
Nerve invasion	455				
No	292	Reference			
Yes	163	1.077 (0.681 – 1.687)	0.748		
Vascular tumor thrombus	455				
No	295	Reference			
Yes	160	1.244 (0.789 – 1.945)	0.343		
Ki67	455	0.999 (0.989 – 1.009)	0.867		
Operation time	455	0.999 (0.997 – 1.001)	0.463		
Surgical approach	455				
Lvor-Lewis	127	Reference			
McKeown	296	1.914 (1.131 – 3.367)	0.019		
Transhiatal esophagectomy	32	1.498 (0.539 – 3.806)	0.412	2.863 (0.034 – 193.725)	0.620
Laparoscopic surgery	455				
No	403	Reference		Reference	
Yes	52	0.322 (0.110 – 0.761)	0.019	7.158 (0.087 – 491.926)	0.358

The multivariable logistic regression model was adjusted for age, hypertension, neoadjuvant radiotherapy, CALLY, NLR, PLR and NMR. Bold values indicate statistical significance (P value < 0.05).

As a sensitivity analysis, the penalized logistic regression yielded largely consistent directions of association with the primary multivariable logistic regression model (**Supplementary Table 2**).

**Figure 2:**
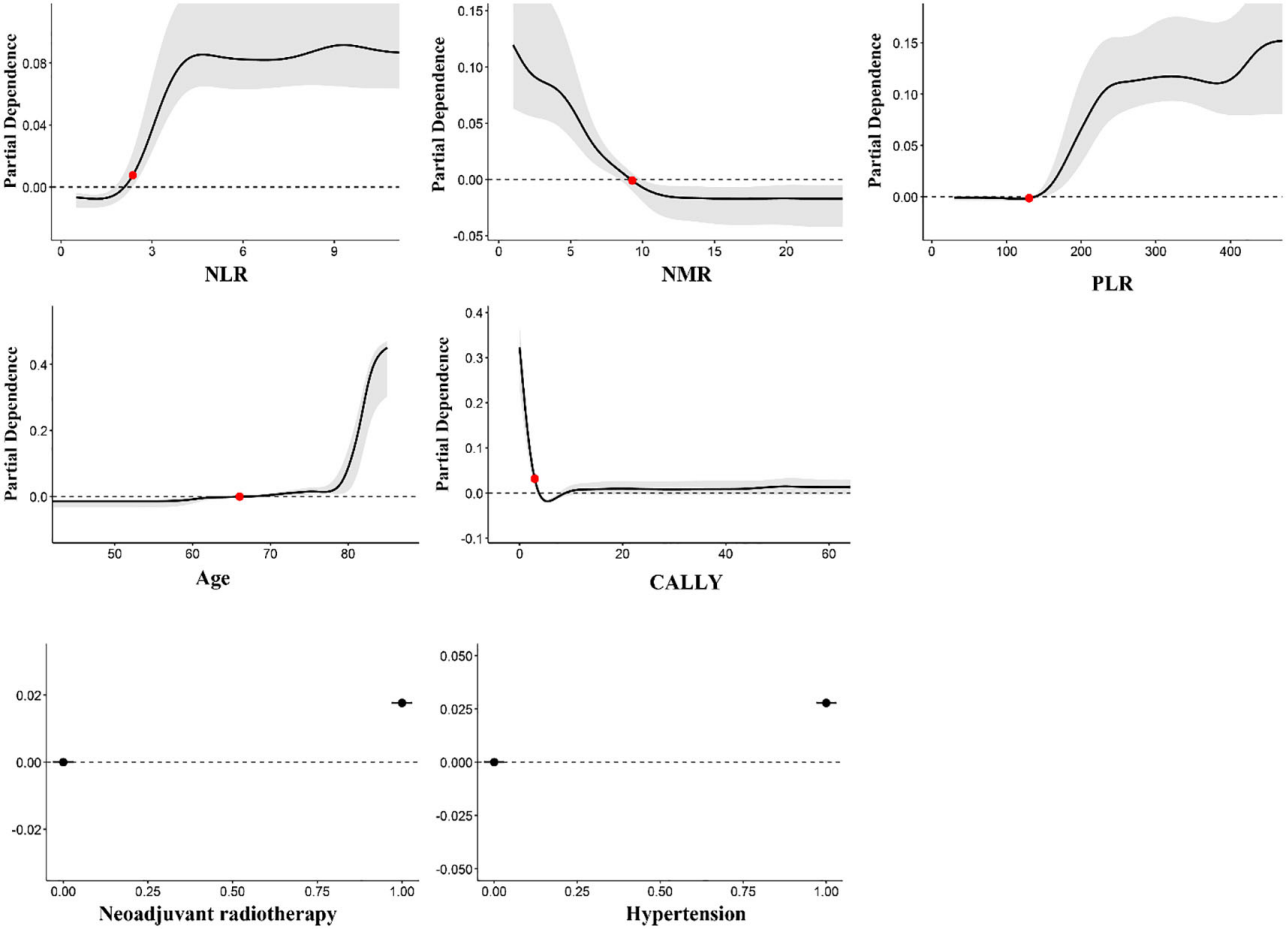
Restricted cubic spline analyses. RCS analyses illustrating the associations between age, CALLY, hypertension, neoadjuvant radiotherapy, NLR, NMR, PLR, and the risk of AL. Solid lines represent adjusted estimates, and shaded areas indicate 95% confidence intervals. Dashed horizontal lines denote the reference level (zero effect). Red dots indicate reference values. Nonlinear relationships were observed for age, inflammatory indices, and nutritional markers, while binary variables showed discrete risk differences.

RCS analysis revealed nonlinear associations between predictors and AL (**Figure 2**). RCS analysis revealed that AL risk increased sharply with age beyond 75 years. For CALLY, risk rose steeply at lower values and plateaued above approximately 1.0, suggesting a threshold effect. Hypertension and neoadjuvant radiotherapy were associated with higher risk. NLR and PLR exhibited positive nonlinear relationships, while NMR showed an inverse association. Violin plot (**Supplementary Figure 2**) analysis demonstrated clear differences in continuous variables between the AL and N-AL groups in the training group. Patients in the AL group were older and exhibited higher NLR and PLR levels, whereas CALLY and NMR values were lower compared with the N-AL group.

### 3.3 Nomogram construction

**Figure 3:**
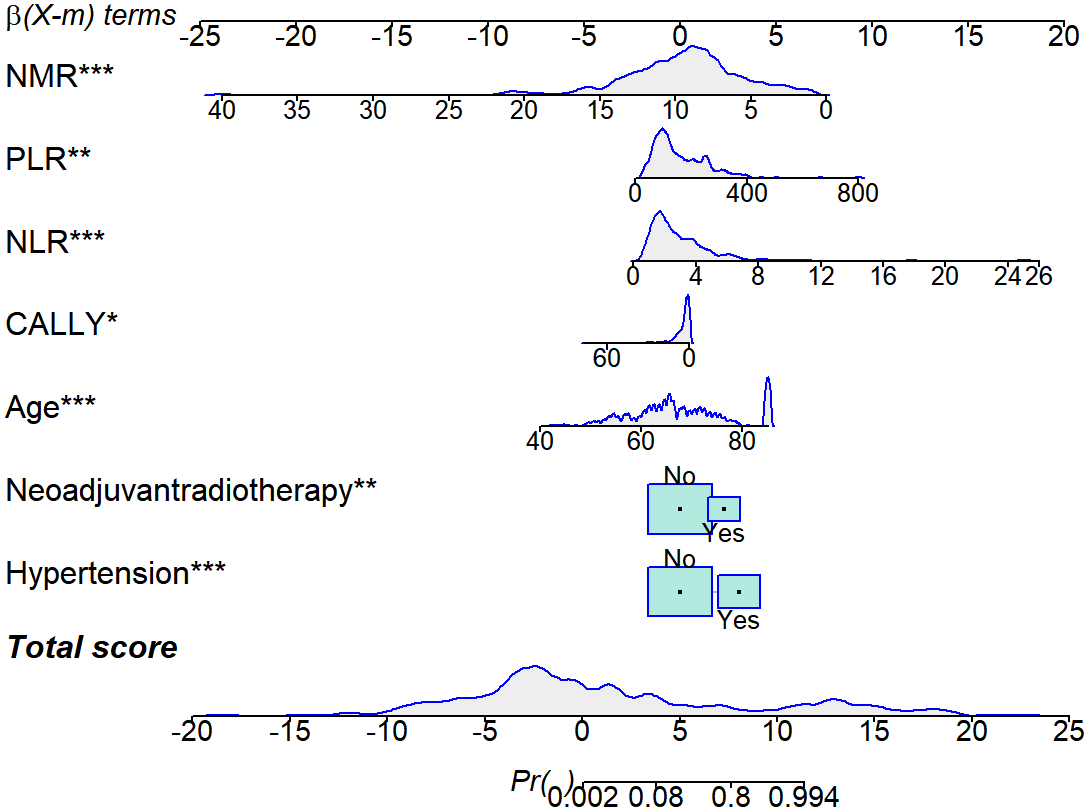
Nomogram for predicting the risk of postoperative AL. Nomogram for predicting the probability of AL based on age, CALLY, NLR, NMR, PLR, hypertension, and neoadjuvant radiotherapy. Each variable corresponds to a weighted score, and the total score is used to estimate individual risk. Asterisks indicate levels of statistical significance.

A nomogram (**Figure 3**) was developed to predict the risk of AL based on the significant predictors identified in the multivariable logistic regression analysis: each predictor was assigned a point range: NMR (0–25 points), PLR (0–15 points), NLR (0–30 points), CALLY (0–10 points), age (0–30 points), neoadjuvant radiotherapy (15 points if yes), and hypertension (25 points if present). The total score is derived by summing the points corresponding to a patient’s values for each predictor.

### 3.4 ROC-derived cutoff values and risk stratification

ROC-derived cutoff values were further calculated for the continuous predictors included in the nomogram. The optimal cutoff values were 78.50 for age, 0.81 for CALLY, 3.04 for NLR, 173.40 for PLR, and 7.09 for NMR, based on the maximum Youden index (**Table 3**). These cutoff values provide clinically interpretable thresholds for identifying patients with a higher risk of AL. In addition, patients were stratified into low-, intermediate-, and high-risk groups according to tertiles of the total nomogram score. The cutoff values and observed AL incidence across the three groups are provided in Supplementary (**Supplementary Table 3**).

**Table 3:** ROC-derived cutoff values for continuous predictors included in the nomogram.

Variable	Cutoff values
Age	78.50
CALLY	0.81
NLR	3.04
PLR	173.40
NMR	7.09

### 3.5 Model performance and validation

**Figure 4:**
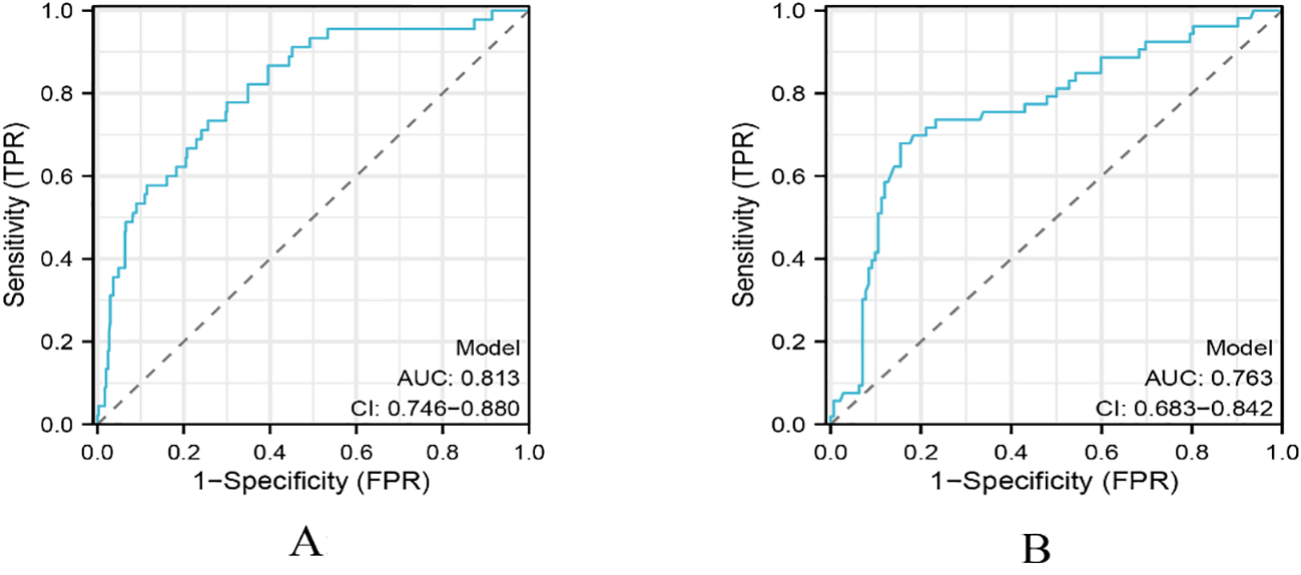
Discrimination of the nomogram model. ROC curves evaluating the discriminative performance of the predictive model in the training **(A)** and validation **(B)** cohorts. The model achieved an AUC of 0.813 (95% CI, 0.746–0.880) in the training group and 0.763 (95% CI, 0.683–0.842) in the validation group. The diagonal dashed line represents the line of no discrimination (AUC = 0.5).

The ROC curves for the training and validation sets were analyzed to evaluate the performance of the predictive model (**Figure 4**). In the training set, AUC was 0.813 (95% CI: 0.746–0.880), indicating good discrimination ability. In the validation set, the AUC was 0.763 (95% CI: 0.683–0.842), demonstrating moderate predictive accuracy.

**Figure 5:**
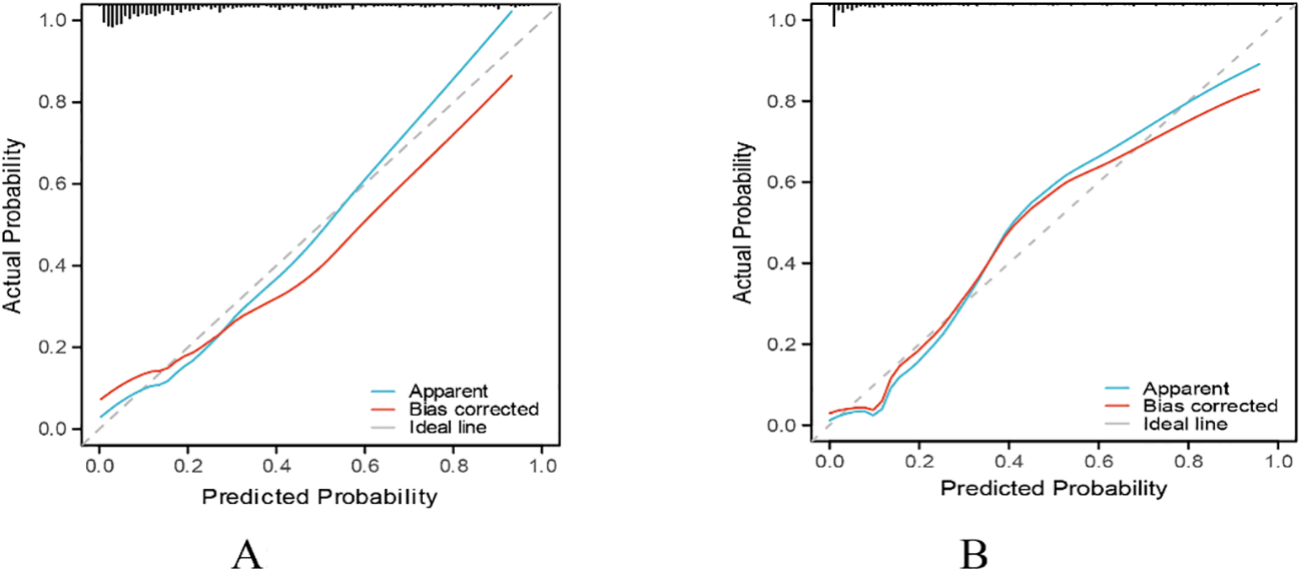
Validation of the nomogram model in the validation cohort. Calibration plots assessing agreement between predicted and observed probabilities in the training **(A)** and validation **(B)** cohorts. The apparent and bias-corrected curves are shown alongside the ideal reference line.

The calibration curves for the training and validation sets were assessed to evaluate the agreement between predicted and actual probabilities of AL (**Figure 5**). In the training set, the calibration curve showed a close fit between the predicted probabilities and actual outcomes, with the bias-corrected line closely following the ideal line, indicating good calibration. Similarly, the calibration curve for the validation set also demonstrated an adequate fit, with the bias-corrected line near the ideal line, confirming the model’s generalizability and reliable prediction of AL risk.

**Figure 6:**
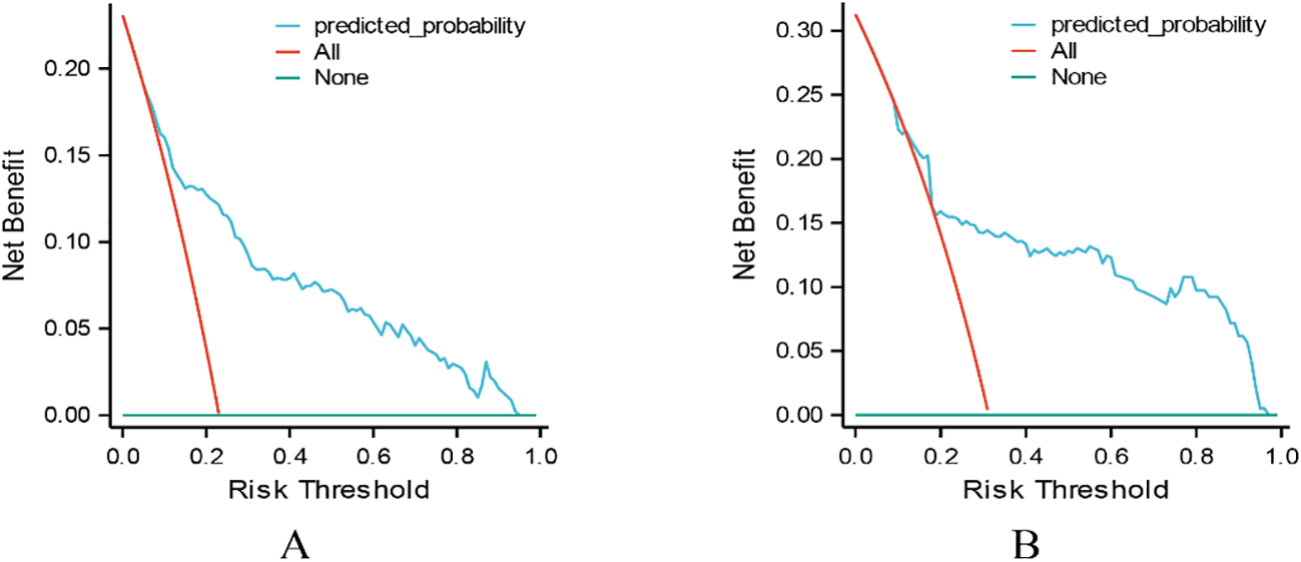
DCA of the nomogram model. DCA plot illustrates the clinical utility of the nomogram in predicting postoperative AL for the training **(A)** and validation cohorts **(B)**. The x-axis denotes the threshold probability, while the y-axis indicates the standardized net benefit. The red curve corresponds to the nomogram model, the gray line represents the scenario in which all patients are assumed to experience the event, and the black line signifies the scenario in which none of the patients are assumed to experience the event.

DCA (**Figure 6**) was performed to evaluate the clinical usefulness of the predictive model in both the training and validation sets. The DCA curves for both sets demonstrated that the model provided a higher net benefit compared to the “All” and “None” strategies across a wide range of risk thresholds. In the training set, the model showed a consistent net benefit, particularly at the risk thresholds between 0.1 and 0.3. Similarly, the DCA for the validation set also indicated that the model maintained a favorable net benefit across various thresholds, with a slight decline at higher thresholds, but still outperformed the “All” and “None” strategies.

## 4 Discussion

AL remains one of the most feared complications after esophagectomy, with reported incidence rates ranging from 5% to 30% depending on the definition criteria, surgical technique, and patient population (1, 2). In the present study, we developed and internally validated a practical nomogram incorporating seven preoperatively available variables—including novel inflammatory-nutritional composite indices—to predict the individual risk of AL after esophagectomy. To our knowledge, this is among the first prediction models that simultaneously integrates the CALLY index with traditional inflammatory markers (NLR, NMR, PLR) and clinical factors (age, hypertension, neoadjuvant radiotherapy), offering a comprehensive assessment of both patient biological vulnerability and treatment context.The incidence of AL in our cohort was 23.5% (153/650), which is consistent with the upper range reported in recent systematic reviews (3, 4). This variation may be attributed to differences in AL definition—our study adopted the ECCG criteria requiring clinical or radiological confirmation (22)—whereas some centers report only severe leaks requiring reoperation. Additionally, the relatively high proportion of advanced-stage disease and the inclusion of both transthoracic and transhiatal approaches in our institution may contribute to this incidence.Consistent with previous reports, we identified age as an independent risk factor for AL (OR 1.319, P < 0.001). Advanced age is associated with diminished physiological reserve, impaired tissue healing, and increased prevalence of comorbidities, all of which compromise anastomotic integrity (5, 6). Notably, restricted cubic spline analysis revealed a sharp increase in risk after 75 years, suggesting a potential threshold effect that warrants consideration in surgical decision-making for elderly patients.Hypertension emerged as a particularly strong risk factor in our model (OR 21.607, P = 0.005), although the wide confidence interval (3.192–284.724) indicates some instability in this estimate. This finding aligns with previous studies demonstrating that chronic hypertension impairs microvascular perfusion and tissue oxygenation, critical factors for anastomotic healing (7, 8). However, the magnitude of this association may reflect unmeasured confounding or interaction with other cardiovascular comorbidities, and should be interpreted with caution.The risk factor of neoadjuvant radiotherapy (OR 16.759, P = 0.026) observed in our study appears compatible with the documented association between radiation-induced tissue fibrosis and impaired wound healing. It should be emphasized that this finding reflects an association within our specific cohort rather than a causal effect. While this result appears to contrast with some reports suggesting that neoadjuvant chemoradiation may facilitate tumor downstaging or technically easier resection in selected patients (9, 12), these discrepancies likely arise from differences in patient selection, radiation protocols, and perioperative management. Thus, this association should be interpreted cautiously and warrants further validation. The wide confidence interval (1.719–297.207) again suggests that this estimate may be unstable, and the association may be influenced by selection bias—patients receiving neoadjuvant therapy may have more favorable tumor characteristics or be treated in higher-volume centers.The incorporation of inflammatory-nutritional indices represents a novel aspect of our model. The CALLY index emerged as a protective factor (OR 0.906, P = 0.044), consistent with its prognostic value in various malignancies. A recent meta-analysis by Allahwala et al. (23) demonstrated that higher CALLY index values were significantly associated with better overall survival (RR 2.24, 95% CI 1.71–2.94, P < 0.001) in esophageal cancer patients. The CALLY index effectively integrates three critical pathophysiological components: systemic inflammation (CRP), nutritional status (albumin), and immune function (lymphocyte count). However, we observed that AL patients had markedly lower CALLY values compared with non-AL patients (median 2.71, IQR 2.70–6.57) in the training cohort; (median1.18, IQR 0.41–3.89) in the validation cohort), which may reflect severe systemic inflammation and malnutrition in this subgroup (17, 23). Similarly, NMR (neutrophil-to-monocyte ratio) demonstrated a strong protective effect (OR 0.369, P < 0.001), while NLR (OR 2.596, P = 0.002) and PLR (OR 1.020, P = 0.007) were risk factors. These findings highlight the complex interplay between different leukocyte subsets in postoperative inflammation. A higher NMR may reflect preserved monocyte-mediated tissue repair mechanisms, whereas elevated NLR and PLR indicate neutrophil-driven inflammation and platelet activation that impair wound healing (24, 25).

Notably, several variables that were significant in univariable analysis—including CAR, and laparoscopic surgery—did not remain independent predictors in the multivariable model. This suggests that their effects may be mediated through other variables in the model or that they interact with other risk factors in complex ways. For instance, the protective effect of laparoscopic surgery observed in univariable analysis (OR 0.322, P = 0.019) was attenuated and reversed in direction in the multivariable model (OR 7.158, P = 0.358), likely due to confounding by indication and the strong effects of other variables (26). The strength of our model lies in its derivation from a large, consecutive cohort with complete follow-up and the rigorous use of restricted cubic splines to capture non-linear effects. The discrimination metrics (C-index 0.813 in training, 0.763 in validation) compare favorably with existing esophageal cancer prediction models. For instance, the nomogram for postoperative pulmonary infection in esophageal cancer patients reported by Li et al. (27) achieved a C-index of 0.802 in the primary cohort and 0.763 in the validation cohort—remarkably similar to our performance. Similarly, the nomogram for postoperative delirium developed by Shen et al. (28) demonstrated AUC values of 0.919 (training) and 0.871 (validation), though this higher performance may reflect the inclusion of postoperative variables. Our model’s advantage lies in its exclusive use of preoperative variables, enabling risk stratification at the time of surgical planning.Moreover, decision curve analysis demonstrated clinical utility across a wide range of threshold probabilities, particularly in the 10%–30% risk range where the net benefit of intervention is maximized. This suggests that surgeons can use the nomogram to stratify patients for targeted interventions—such as intensive blood pressure control, preoperative immunonutrition, or enhanced monitoring—without exposing low-risk patients to unnecessary procedures.Finally, ROC-derived cutoff values and tertile-based stratification of the total nomogram score may provide practical tools for individualized perioperative risk assessment. However, these cutoff values and risk strata were derived from the present cohort and require further validation in external populations.

Several limitations merit consideration, along with corresponding mitigation strategies and future directions. First, our study included patients over a ten-year period (2015–2025), during which perioperative management of esophageal cancer underwent significant changes, including the widespread adoption of minimally invasive surgical techniques, advancements in nutritional support, improvements in intensive care, and the evolution of the surgical learning curve (29, 30). Although it is difficult for a retrospective study to adjust for all these variables, we did adjust for laparoscopic surgery and year of surgery, and performed sensitivity analyses to further confirm the robustness of our model. These results suggest that the change in calendar year may have only a minor impact on AL. Future studies are needed to validate the time effect in more complex real-world settings (31). Several inflammatory indices may reflect related biological pathways, and potential overlap among these markers should be acknowledged. Nevertheless, the overall pattern of associations remained generally stable in the additional regularization analyses.

Second, external validity remains to be established. To address this, collaborative multicenter validation studies are warranted to test the model’s performance across diverse healthcare settings, surgical techniques, and patient populations. Such efforts would enhance generalizability and potentially lead to the development of a universally applicable risk calculator.Third, the wide confidence intervals observed for some covariates, particularly hypertension and neoadjuvant radiotherapy, suggest statistical uncertainty in effect size estimation, likely due to imbalanced subgroup distributions and limited event counts within specific strata. As a result, these findings primarily support the presence of an association rather than precise quantification of effect magnitude. The Firth penalized regression produced findings that were broadly consistent with the primary multivariable model, with narrower confidence intervals observed for several predictors. This further supports the robustness of the identified associations. Therefore, these effect estimates should be interpreted cautiously, with greater emphasis placed on the direction and robustness of the associations rather than the exact magnitude of risk. Future studies with larger sample sizes and more balanced subgroup distributions are needed to refine these estimates and improve precision.Fourth, detailed information on specific anastomotic techniques was not consistently available in our retrospective database. Nevertheless, other operative factors, such as surgical approach and laparoscopic versus open approach, were included in the analysis. While the absence of these specific variables may slightly limit the ability to fully explore their impact on AL, the model still incorporates key clinical and operative factors and provides meaningful predictive performance. Future studies with more detailed surgical data could further enhance the model’s precision and applicability.

Finally, this was a retrospective study based on an institutional database. Due to incomplete availability of some historical medical records during the early study period, a proportion of cases were identified through alternative institutional sources, which may have introduced a degree of selection bias. Consequently, the observed incidence of AL in the present cohort may differ from that reported in continuous, unselected populations. While such bias could potentially influence absolute risk estimates, the discrimination performance of the model remained acceptable in both the training and validation cohorts. Future prospective studies based on consecutive patient enrollment are warranted to further validate the model. Moreover, residual confounding by unmeasured factors—such as conduit ischemia time, surgeon volume, or intraoperative hypotension—cannot be ruled out. To address this, prospective data collection protocols should incorporate these technical variables, and future model iterations could include intraoperative data to enhance predictive accuracy. Additionally, the integration of radiomic features from pre-operative CT or PET images may capture conduit vascularity and local inflammation beyond serum biomarkers (32), representing a promising avenue for model enhancement.

In conclusion, the proposed nomogram provides an individualized, biologically informed estimate of AL risk and may serve as a practical tool for shared decision-making, resource allocation, and the design of risk-adapted trials aimed at reducing esophagectomy-related morbidity. The identification of age, hypertension, and inflammatory markers as key risk factors provides actionable targets for preoperative optimization and patient counseling.

## Data Availability

The original contributions presented in the study are included in the article/**Supplementary Material**. Further inquiries can be directed to the corresponding authors.

## References

[1] Yang H . Oesophageal cancer. Lancet. (2024) 404:1991–2005. doi: 10.1016/s0140-6736(24)02226-8 39550174

[2] Jiang W . Current status and perspectives of esophageal cancer: a comprehensive review. Cancer Commun (Lond). (2025) 45:281–331. doi: 10.1002/cac2.12645 39723635 PMC11947622

[3] Fabbi M . Anastomotic leakage after esophagectomy for esophageal cancer: definitions, diagnostics, and treatment. Dis Esophagus. (2021) 34:doaa039. doi: 10.1093/dote/doaa039 32476017 PMC7801633

[4] Zhong Y Sun R Li W Wang W Che J Ji L . Risk factors for esophageal anastomotic stricture after esophagectomy: a meta-analysis. BMC Cancer. (2024) 24:872. doi: 10.1186/s12885-024-12625-8 39030531 PMC11264988

[5] Triantafyllou A Mela E Theodoropoulos C Theodorou AP Kitsou E Saliaris K . Addressing anastomotic leak after esophagectomy: insights from a specialized unit. J Clin Med. (2025) 14:3694. doi: 10.3390/jcm14113694 40507455 PMC12156875

[6] Kamarajah SK . Navigating complexities and considerations for suspected anastomotic leakage in the upper gastrointestinal tract: a state of the art review. Best Pract Res Clin Gastroenterol. (2024) 70:101916. doi: 10.1016/j.bpg.2024.101916 39053974

[7] Shao S Li Y Cheng H Chen C Zeng Y Huang W . Prospective multicenter validation of a machine learning model for predicting anastomotic leakage in patients with gastric adenocarcinoma undergoing total or proximal gastrectomy. Int J Surg. (2025) 111:8027–36. doi: 10.1097/js9.0000000000003025 40696942 PMC12626478

[8] Tverskov V . The impact of cervical anastomotic leak after esophagectomy on long-term survival of patients with esophageal cancer. Surgery. (2022) 171:1257–62. doi: 10.1016/j.surg.2021.10.011 34750016

[9] Seo HW Jeon YJ Cho JH Kim HK Choi YS Zo JI . Treatment patterns and outcomes of anastomotic leakage after esophagectomy for esophageal cancer. J Chest Surg. (2024) 57:152–9. doi: 10.5090/jcs.23.114 38228498 PMC10927423

[10] Low DE Kuppusamy MK Alderson D Cecconello I Chang AC Darling G . Benchmarking complications associated with esophagectomy. Ann Surg. (2019) 269:291–8. doi: 10.1097/sla.0000000000002611 29206677

[11] Low DE Alderson D Cecconello I Chang AC Darling GE D'Journo XB . International consensus on standardization of data collection for complications associated with esophagectomy: esophagectomy complications consensus group (ECCG). Ann Surg. (2015) 262:286–94. doi: 10.1097/sla.0000000000001098 25607756

[12] Hu Q Sun L Xu S Hong W Tang L Li F . Analysis of risk factors for anastomotic leakage after radical esophagectomy for esophageal squamous cell carcinoma. Front Med (Lausanne). (2025) 12:1668790. doi: 10.3389/fmed.2025.1668790 41244783 PMC12615427

[13] Tamai K Okamura S Makino S Yamamura N Fukuchi N Ebisui C . C-reactive protein/albumin ratio predicts survival after curative surgery in elderly patients with colorectal cancer. Updates Surg. (2022) 74:153–62. doi: 10.1007/s13304-021-01011-9 33677820

[14] van Hagen P Hulshof MC van Lanschot JJ Steyerberg EW van Berge Henegouwen MI Wijnhoven BP . Preoperative chemoradiotherapy for esophageal or junctional cancer. N Engl J Med. (2012) 366:2074–84. doi: 10.1056/nejmoa1112088 22646630

[15] Li M Li Z Wang Z Yue C Hu W Lu H . Prognostic value of systemic immune-inflammation index in patients with pancreatic cancer: a meta-analysis. Clin Exp Med. (2022) 22:637–46. doi: 10.1007/s10238-021-00785-x 35022918

[16] Tian BW Yang YF Yang CC Yan LJ Ding ZN Liu H . Systemic immune-inflammation index predicts prognosis of cancer immunotherapy: systemic review and meta-analysis. Immunotherapy. (2022) 14:1481–96. doi: 10.2217/imt-2022-0133 36537255

[17] Ma R Okugawa Y Shimura T Yamashita S Sato Y Yin C . Clinical implications of C-reactive protein-albumin-lymphocyte (CALLY) index in patients with esophageal cancer. Surg Oncol. (2024) 53:102044. doi: 10.1016/j.suronc.2024.102044 38335851

[18] Sugimoto A Toyokawa T Miki Y Yoshii M Tamura T Sakurai K . Preoperative C-reactive protein to albumin ratio predicts anastomotic leakage after esophagectomy for thoracic esophageal cancer: a single-center retrospective cohort study. BMC Surg. (2021) 21:348. doi: 10.1186/s12893-021-01344-7 34548054 PMC8454123

[19] Zhou C Ma G Li X Li J Yan Y Liu P . Is minimally invasive esophagectomy effective for preventing anastomotic leakages after esophagectomy for cancer? a systematic review and meta-analysis. World J Surg Oncol. (2015) 13:269. doi: 10.1186/s12957-015-0661-z 26338060 PMC4560054

[20] Briez N Piessen G Bonnetain F Brigand C Carrere N Collet D . Open versus laparoscopically-assisted oesophagectomy for cancer: a multicentre randomised controlled phase III trial - the MIRO trial. BMC Cancer. (2011) 11:310. doi: 10.1186/1471-2407-11-310 21781337 PMC3156811

[21] Frasson M Flor-Lorente B Rodríguez JL Granero-Castro P Hervás D Alvarez Rico MA . Risk factors for anastomotic leak after colon resection for cancer: multivariate analysis and nomogram from a multicentric, prospective, national study with 3193 patients. Ann Surg. (2015) 262:321–30. doi: 10.1097/sla.0000000000000973 25361221

[22] Low DE Alderson D Cecconello I Chang AC Darling GE D'Journo XB . International consensus on standardization of data collection for complications associated with esophagectomy for esophageal cancer: the esophagectomy complications consensus group (ECCG). Ann Surg. (2015) 262:286–94. doi: 10.1097/sla.0000000000001098 25607756

[23] Ul Bassar W Akhtar J Mukhtar Ismail NH Shah Z Kaur Dhanjal M Chaudhary S . The C-reactive protein-albumin-lymphocyte index predicts survival outcomes in esophageal cancer: a meta-analysis. Cureus. (2025) 17:e93594. doi: 10.7759/cureus.93594 41179090 PMC12574980

[24] Pirozzolo G Gisbertz SS Castoro C van Berge Henegouwen MI Scarpa M . Neutrophil-to-lymphocyte ratio as a prognostic marker in esophageal cancer: a systematic review and meta-analysis. J Thorac Dis. (2019) 11:3136–45. doi: 10.21037/jtd.2019.07.30 PMC6688029 31463142

[25] Zhang R . The C-reactive protein/albumin ratio predicts poor survival and correlates with systemic inflammatory response in nasopharyngeal carcinoma. Oncotarget. (2016) 7:43925–33. doi: 10.1007/s11605-019-04495-4

[26] Zheng H Wu Z Wu Y Mo S Dai W Liu F . Laparoscopic surgery may decrease the risk of clinical anastomotic leakage and a nomogram to predict anastomotic leakage after anterior resection for rectal cancer. Int J Colorectal Dis. (2019) 34:319–28. doi: 10.1007/s00384-018-3199-z 30470941 PMC6331738

[27] Li J . A nomogram for predicting postoperative pulmonary infection in esophageal cancer patients. BMC Pulm Med. (2021) 21:281. doi: 10.1186/s12890-021-01656-7 34488717 PMC8422704

[28] Shen X Yang L Jiang L Wang Q Liu YY Song SZ . Development and validation of a nomogram model to predict postoperative delirium among esophageal cancer resection patients. Sci Rep. (2025) 15:11255. doi: 10.1038/s41598-025-11255-9 40691213 PMC12280112

[29] Biere SS van Berge Henegouwen MI Maas KW Bonavina L Rosman C Garcia JR . Minimally invasive versus open oesophagectomy for patients with oesophageal cancer: a multicentre, open-label, randomised controlled trial. Lancet. (2012) 379:1887–92. doi: 10.1016/s0140-6736(12)60516-9 22552194

[30] Luketich JD Pennathur A Awais O Levy RM Keeley S Shende M . Outcomes after minimally invasive esophagectomy: review of over 1000 patients. Ann Surg. (2012) 256:95–103. doi: 10.1097/SLA.0b013e3182590603 PMC4103614 22668811

[31] Briez N Piessen G Bonnetain F Brigand C Carrere N Collet D . Open versus laparoscopically-assisted oesophagectomy for cancer: a multicentre randomised controlled phase III trial (the MIRO trial). Lancet. (2019) 393:1035–43. doi: 10.1186/1471-2407-11-310 PMC3156811 21781337

[32] Ji X . CT-radiomics combined with inflammatory indicators for prediction of progression free survival of resectable esophageal squamous cell carcinoma. Sci Rep. (2025) 15:1240. doi: 10.1038/s41598-025-01240-7 40348786 PMC12065806

