# Supplementary material for: Prediction of anastomotic leakage after esophagectomy for esophageal cancer: a nomogram study integrating systemic inflammation indices and clinical factors
Source: Front Oncol. 2026 Jul 14;16:1821694. doi: 10.3389/fonc.2026.1821694 (PMC13407267; doi:10.3389/fonc.2026.1821694)
Supplement: Supplementary file 1 [file DataSheet1.docx]

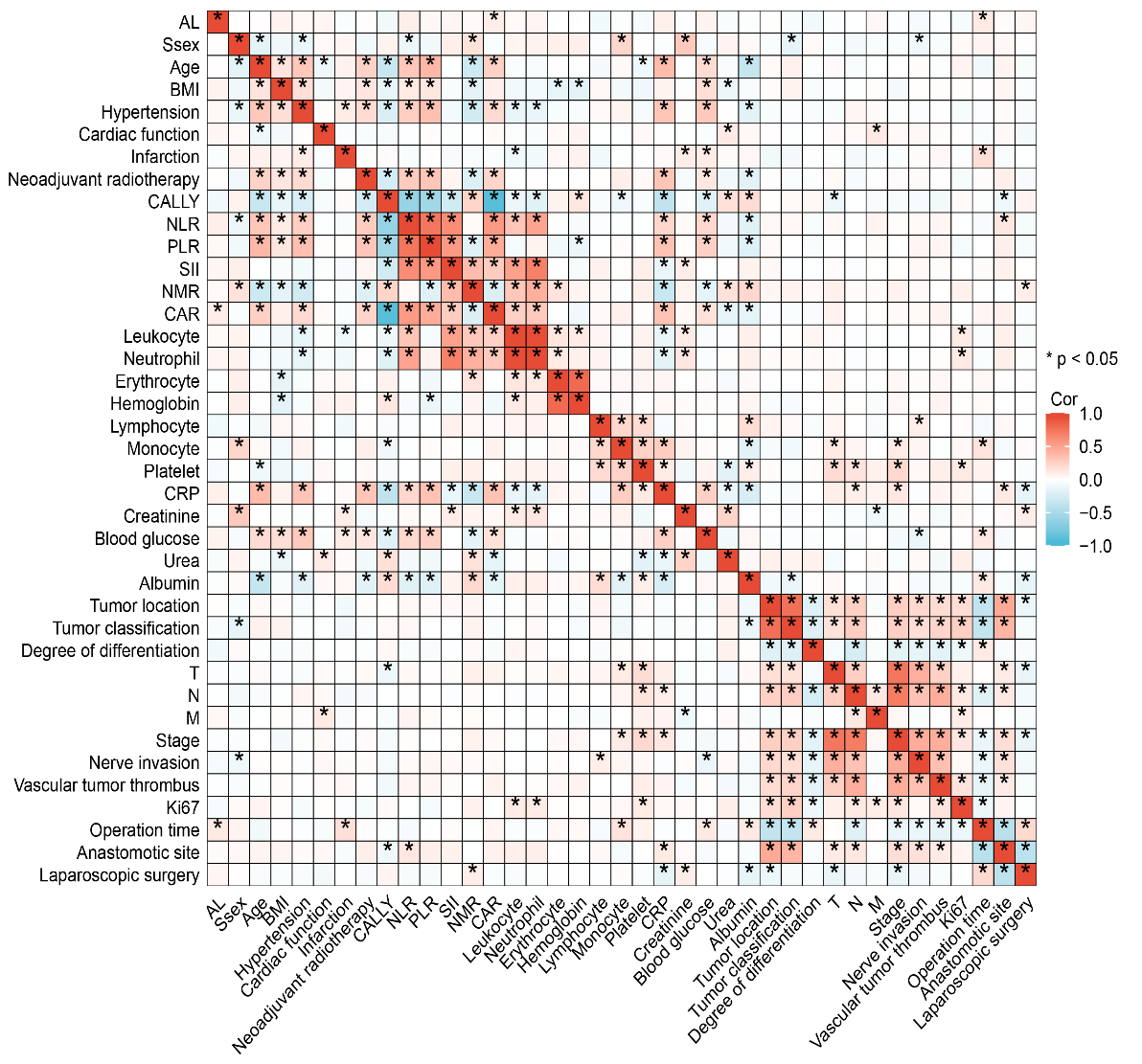


**Figure. S1： Correlation Matrix of Clinicopathological and Laboratory Variables**

Heatmap illustrating pairwise correlations among demographic, clinicopathological, laboratory, and surgical variables, including AL. Color intensity represents the magnitude and direction of correlation (red, positive; blue, negative). Asterisks denote statistically significant correlations (*P < 0.05).


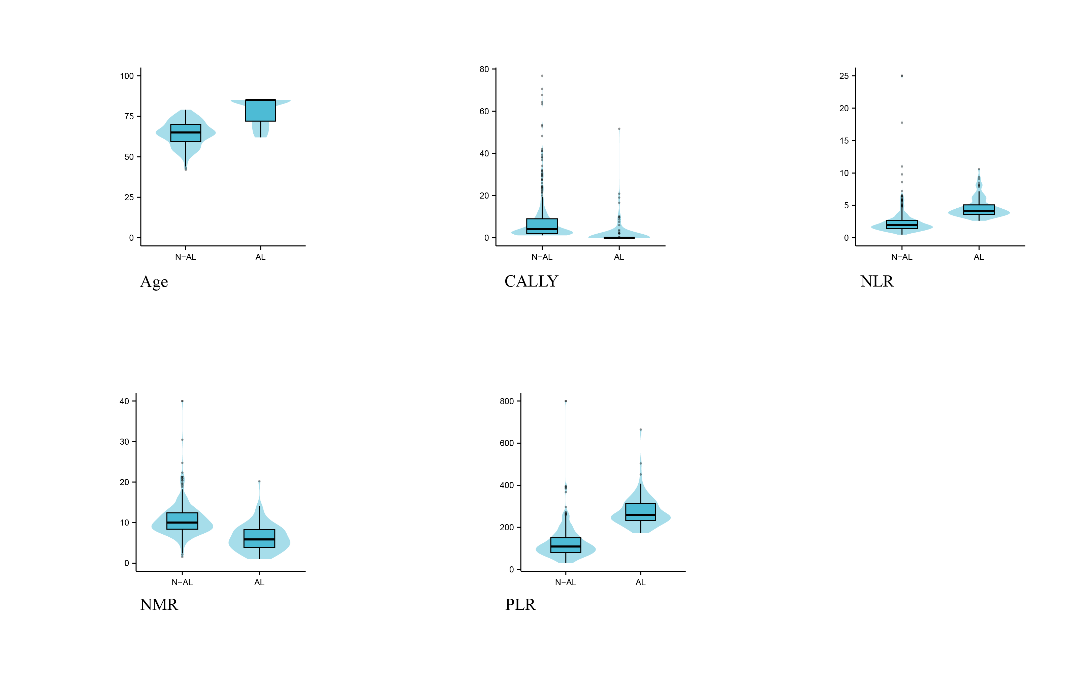


**Figure. S2： Violin Plots Showing the Distribution of Age, NMR, CALLY, NLR, and PLR in the Study Cohort.**

Violin plots depicting the distribution of five continuous variables (Age, NMR, CALLY, NLR, and PLR) across the study population. Each plot represents the probability density of the data, with the white dot indicating the median, the thick black bar representing the interquartile range, and the thin black line extending to the non-outlier range.

**Table S1. Variance Inflation Factor (VIF) for Predictor Variables in multivariate Logistic Regression.**

| **Variables** | **Types** | **VIF** |
| --- | --- | --- |
| Hypertension | Ordinal Variable |  |
| 0 |  | Reference |
| 1 |  | 1.4952 |
| Neoadjuvant radiotherapy | Ordinal Variable |  |
| 0 |  | Reference |
| 1 |  | 1.2193 |
| CALLY | Continuous Variable | 1.1294 |
| NLR | Continuous Variable | 7.1014 |
| PLR | Continuous Variable | 3.6924 |
| Age | Continuous Variable | 1.7298 |
| NMR | Continuous Variable | 6.5427 |

**Table S2. Firth Penalized Logistic Regression Analysis of Predictors for Anastomotic Leakage.**

| Variable | OR (95% CI) | P-value |
| --- | --- | --- |
| Age | 13.11 (4.24-65.16) | <0.001 |
| Hypertension | 18.93 (3.97-154.43) | <0.001 |
| Neoadjuvant.radiotherapy | 6.67 (1.33-46.13) | 0.021 |
| CALLY | 0.32 (0.13-0.67) | 0.002 |
| NLR | 12.81 (3.28-94.04) | <0.001 |
| PLR | 1.95 (0.57-11.80) | 0.32 |
| NMR | 0.08 (0.02-0.23) | <0.001 |

[Note]: The Firth penalized logistic regression model was adjusted for age, hypertension, neoadjuvant radiotherapy, CALLY, NLR, PLR, NMR, and calendar year.

**Table S3. Nomogram-based risk stratification according to total score tertiles.**

| Risk group | Total nomogram score | Total, n |
| --- | --- | --- |
| Low-risk | <38.90 | 152 |
| Intermediate-risk | 38.90 to <51.03 | 151 |
| High-risk | ≥51.03 | 152 |
